# Successful Treatment of Mesenteric Varices by Retrograde Transvenous Obliteration by the Delivery of *N*-butyl-2-cyanoacrylate via an Abdominal Wall Vein

**DOI:** 10.1007/s00270-013-0647-6

**Published:** 2013-05-29

**Authors:** Osamu Ikeda, Yutaka Nakasone, Koichi Yokoyama, Seijiro Inoue, Hiroshi Takamori, Hideo Baba, Yasuyuki Yamashita

**Affiliations:** 1 Department of Diagnostic Radiology, Kumamoto University Graduate School of Medical and Pharmaceutical Sciences, 1-1-1, Honjo, Kumamoto, 860-8505 Japan; 2 Department of Gastroenterological Surgery, Kumamoto University Graduate School of Medical and Pharmaceutical Sciences, 1-1-1, Honjo, Kumamoto, 860-8505 Japan

## Abstract

Bleeding from mesenteric varices associated with portal hypertension is occasionally life-threatening. A 53-year-old man who had undergone esophageal transection for esophageal varices and balloon-occluded retrograde transvenous obliteration for gastric varices presented with melena due to ruptured mesenteric varices. He was treated by injecting *N*-butyl-2-cyanoacrylate via an abdominal wall vein to obtain retrograde transvenous obliteration.

## Introduction

Ectopic varices outside the gastroesophageal region have been reported in patients with portal hypertension. According to Lebrec and Benhamou [1], as many as one-third of mesenteric varices are seen in the jejunum and ileum. Patients with chronic liver disease can manifest different pathways of portosystemic shunts and these can result in gastroesophageal bleeding [2]. Gastric variceal bleeding is usually treated by endoscopic obliteration with *N*-butyl-2-cyanoacrylate (NBCA; Histoacryl, B. Braun, Melsungen, Germany) [3]. Balloon-occluded retrograde transvenous obliteration (BRTO) is a widely accepted treatment for patients with ectopic varices. In our patient, we identified a remarkably tortuous, small draining vein functioning as a collateral vein from the mesenteric varices to an abdominal wall vein on contrast-enhanced CT scans and in the venous phase of superior mesenteric arteriography. Therefore, we adopted retrograde transvenous obliteration (RTO) instead of BRTO by inserting an 18-gauge plastic needle in an abdominal wall vein after surgical incision. We present this unusual case whose mesenteric varices were successfully treated by RTO using *N*-butyl-2-cyanoacrylate.

## Case Report

Our interventional protocol and retrospective study were approved by the Human Subjects Research Review Board and patient consent was waived. A 53-year-old man with hepatocellular carcinoma suffered chronic hepatitis due to hepatitis B virus infection since undergoing esophageal transection for esophageal- and BRTO for gastric varices. He had been treated previously by three transcatheter arterial chemoembolizations and two percutaneous radiofrequency ablations. He was anemic due to melena and started suffering abdominal pain approximately 1 week earlier. Blood transfusions failed to improve his symptoms. On the day of the procedure, he manifested hematemesis. His heart rate ranged from 70 to 80 beats/min. His systolic blood pressure averaged 60–80 mmHg. Laboratory examination revealed anemia (red blood cell count 1.77 × 10^6^/μl, hemoglobin 4.0 mg/dl); his liver function was graded as Child-Pugh B (total bilirubin 0.6 mg/dl, albumin 2.6 g/dl, prothrombin time 62 %, ascites).

**Fig. 1:**
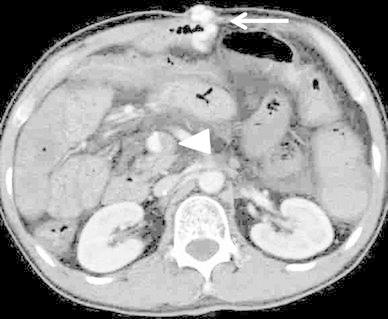
Contrast-enhanced CT scan reveals mesenteric varices (*arrow*) feeding the abdominal wall vein. All varices were directly connected. There is thrombosis in the mesenteric vein (*arrowhead*)

No active bleeding was identified by esophagogastroduodenoscopy and colonoscopy. Mesenteric variceal rupture into the small intestine was suspected because on contrast-enhanced CT scans, we observed mesenteric varices via small abdominal wall veins and thrombosis in the superior mesenteric vein (Fig. 1). On fusion images of SPECT using 99mTc-pyrophosphate (370 MBq) and nonenhanced helical CT images, a dense accumulation of mesenteric varices was seen. Conservative management with fasting did not improve his progressive anemia or melena. We obtained his prior written, informed consent for angiography and RTO using NBCA or 5 % ethanolamine oleate (EOI) and for inclusion in our study.

**Fig. 2:**
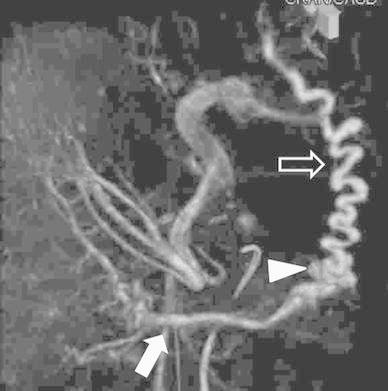
Superior mesenteric arteriography (cone beam CT, venous phase) demonstrates mesenteric varices (*arrowhead*) from the jejunal vein (*arrow*) to a draining vein in the abdominal wall (*open arrow*)

**Fig. 3:**
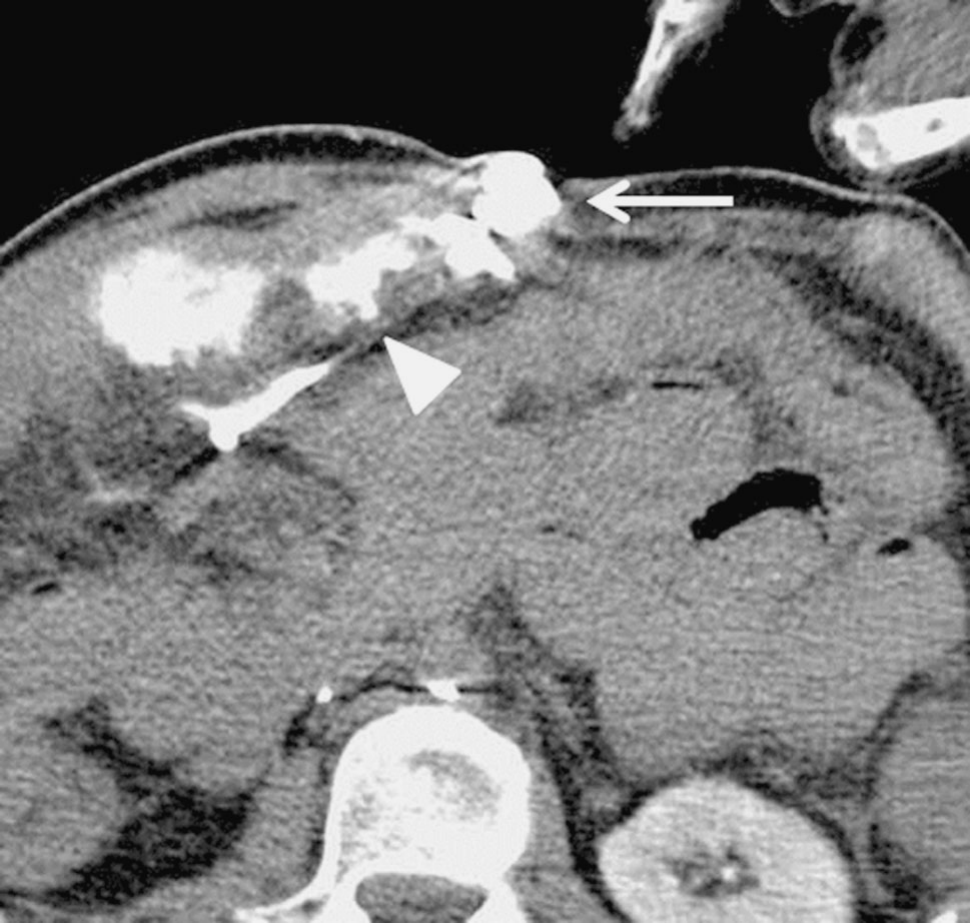
Retrograde CT images through the 18-gauge needle obtained by manually injecting contrast medium show mesenteric varices (*arrow*) and extravasation into the small bowel (*arrowhead*)

**Fig. 4:**
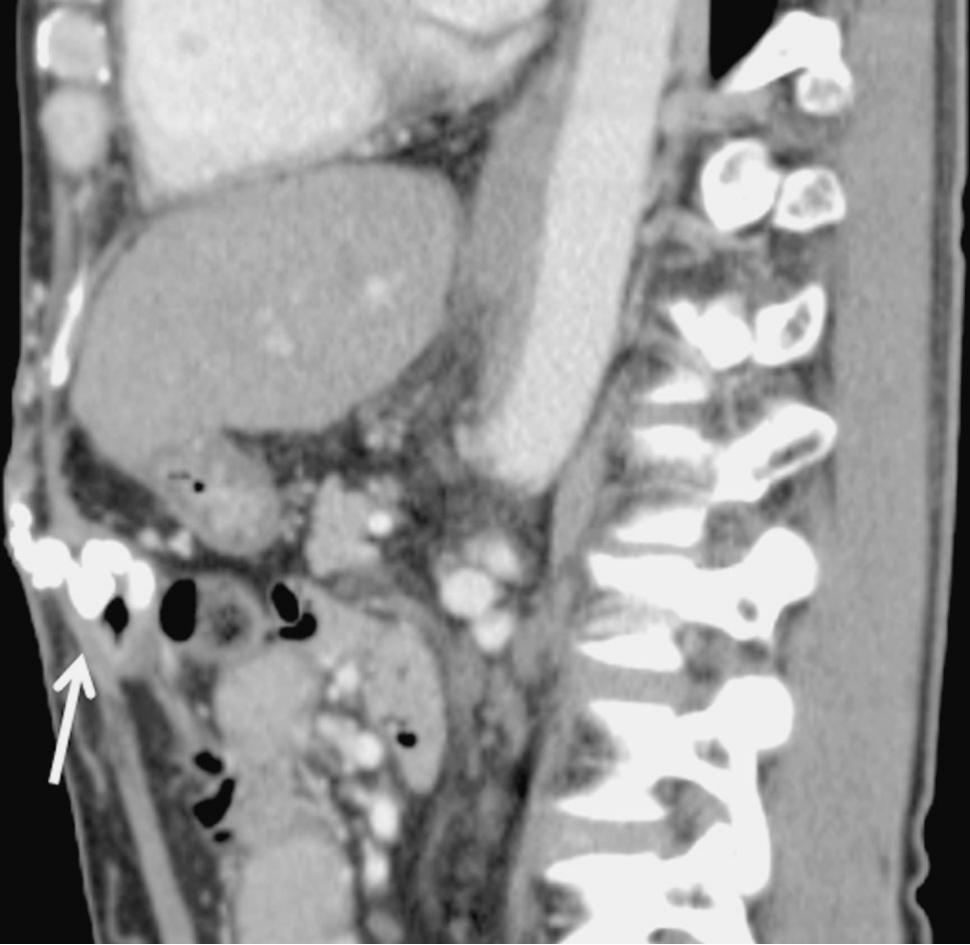
CT scan reveals the retention of the NBCA mixture in the mesenteric varices (*arrow*). Note the draining vein observed just after retrograde transvenous obliteration

In the venous phase of superior mesenteric arteriography, we observed a collateral vein from the mesenteric varices to small abdominal wall veins (Fig. 2). We performed a surgical incision, and after inserting an 18-gauge plastic needle into an exposed abdominal wall vein we ligated a distal abdominal wall vein. We obtained retrograde CT images through the 18-gauge needle by manually injecting 5 ml of contrast medium (Iopamiron300; Schering Japan, Osaka) to visualize the mesenteric varices and extravasation into the small bowel (Fig. 3). The sclerosing agent was a 1:4 mixture of NBCA and iodized oil (Lipiodol; Laboratoire Guerbet, Roissy, France); this yielded radiopacity. The mixture of NBCA and lipiodol (1 ml) was injected slowly under fluoroscopic monitoring until the mesenteric varices were completely filled. The procedure was terminated when a CT scan acquired just after sclerotherapy demonstrated complete coagulation (Fig. 4).

**Fig. 5:**
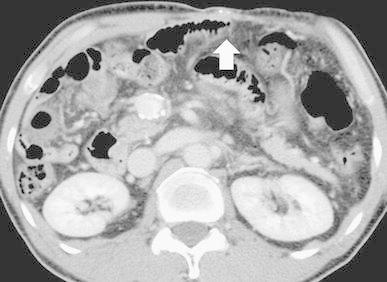
CT scan shows spotty retention of the NBCA mixture in the draining vein in the abdominal wall (*arrow*). There is no recurrence of mesenteric varices (*arrow*)

By these procedures, his melena improved and hemoglobin level increased from 3.6 to 10.2 mg/dl. He was discharged on the tenth day after sclerotherapy. During the course of 1-year follow-up, his esophageal varices worsened and he underwent endoscopic variceal ligation 6 months after the procedure. He suffered no hemorrhages by ectopic varices and his hemoglobin level did not decrease in the follow-up period (Fig. 5).

## Discussion

The incidence of small-bowel varices is low. Bleeding from ectopic varices is reported in 1–5 % of patients with liver cirrhosis and in 20–30 % of patients with extrahepatic portal hypertension [1, 4]. Of bleeding ectopic varices, 17 % each were found in the duodenum, jejunum, or ileum, 14 % in the colon, 8 % in the rectum, and 9 % in the peritoneum [5]. Roughly half of patients with ectopic variceal bleeding had a history of surgery [4]. In fact, 66 % of patients with small-bowel varices had undergone abdominal or pelvic surgery, suggesting that postoperative adhesions between an incision in the abdominal wall and the small bowel may result in the formation of collaterals drained by the systemic venous circulation [1]. In our patient, the mesenteric varices were attributable to portal hypertension and postoperative adhesions.

Therapeutic options for bleeding ectopic varices include local treatment for the varices and portal decompression. Local interventional radiology includes percutaneous transhepatic obliteration (PTO) and BRTO. Portal decompressive treatment includes surgical dissection and transjugular intrahepatic portosystemic shunt (TIPS). L’Hermine et al. [6] reported that PTO controlled bleeding from gastroesophageal varices in 83 % of their patients. However, the formation of new inflow routes may result in rebleeding after PTO [5, 7]. According to Ninoi et al. [8], BRTO more effectively controls gastric variceal bleeding than TIPS or PTO.

Ono et al. [9] reported the successful management of mesenteric varices with BRTO performed via an abdominal wall collateral vein; they accessed the vein percutaneously under real-time ultrasound guidance. Ikeda et al. [10] addressed mesenteric varices with RTO via the abdominal wall. They accessed the vein after surgical incision.

We identified a remarkably tortuous small draining vein functioning as a collateral vein from the mesenteric varices to an abdominal wall vein on contrast-enhanced CT images and in the venous phase of superior mesenteric arteriography. Therefore, we adopted RTO instead of BRTO by inserting an 18-gauge plastic needle in an abdominal wall vein after surgical incision.

Various sclerosants and tissue adhesives have been employed for the management of duodenal variceal bleeding. Hirota et al. [11] used an EOI mixture or endoscopic injection sclerotherapy at BRTO. EOI agglutinates platelets and destroys vascular endothelial cells; consequently, it functions as a sclerotic agent. To obtain sclerosis of mesenteric varices, the sclerosing agent must remain in situ for at least 1 h [11]. Ikeda et al. [10] reported that manual pressure applied to the abdominal wall keeps the injected EOI in the varices for 1 h. NBCA, a tissue adhesive that rapidly polymerizes upon contact with blood and embolizes the varices, has been used to achieve hemostasis in patients with gastrointestinal variceal bleeding [12]. In our patient, we observed remarkable extravasation into the small bowel as we performed retrograde venography through an abdominal wall vein by manually injecting the contrast medium. Therefore, we used the sclerosant with NBCA to obtain rapid embolization of the varices. This achieved a favorable outcome.

In the short-term follow-up period, our patient’s esophageal varices worsened and he underwent endoscopic variceal ligation 6 months after the initial procedure. He did not develop hemorrhages from ectopic varices, although the obliteration of a collateral vein can induce the development of other varices. Therefore, we will continue careful follow-up for the long-term.

In summary, we report a case of bleeding mesenteric varices controlled by RTO using NBCA delivered via an abdominal wall vein and found that this technique can be selected for treatment of postoperative mesenteric varices.

## References

[1] Lebrec D, Benhamou J (1985). Ectopic varices in portal hypertension. Clin Gastroenterol.

[2] Ibukuro K, Sugihara T, Tanaka R (2007). Balloon-occluded retrograde transvenous obliteration (BRTO) for a direct shunt between the inferior mesenteric vein and the inferior vena cava in a patient with hepatic encephalopathy. J Vasc Radiol.

[3] Kang EJ, Jeason SW, Jang JY (2011). Long-term result of endoscopic histoacryl (*N*-butyl-2-cyanoacrylate) injection for treatment of gastric varices. World J Gastroenterol.

[4] Heaton ND, Khawaja H, Howard ER (1991). Bleeding duodenal varices. Br J Surg.

[5] Norton ID, Andrews JC, Kamath PS (1998). Management of ectopic varices. Hepatology.

[6] L’Hermine C, Chastanet P, Delemazure O (1989). Percutaneous transhepatic embolization of gastroesophageal varices: results in 400 patients. AJR Am J Roentgenol.

[7] Chikamori F, Kuniyoshi N, Kagiyama S (2007). Role of percutaneous transhepatic obliteration for special types of varices with portal hypertension. Abdom Imaging.

[8] Ninoi T, Nakamura K, Kaminou T (2004). TIPS versus transcatheter sclerotherapy for gastric varices. Am J Roentgenol.

[9] Ono S, Irie T, Kuramochi M (2007). Successful treatment of mesenteric varices with balloon-occluded retrograde transvenous obliteration via an abdominal wall vein. J Vasc Interv Radiol.

[10] Ikeda O, Tamura Y, Nakasone Y (2010). Successful treatment of mesenteric varices after living donor liver transplantation with retrograde transvenous obliteration via an abdominal wall vein. Cardiovasc Intervent Radiol.

[11] Hirota S, Matsumoto S, Sako M (1999). Retrograde transvenous obliteration of gastric varices. Radiology.

[12] Marques P, Maluf-Filho F, Kumar A (2008). Long-term outcomes of acute gastric variceal bleeding in 48 patients following treatment with cyanoacrylate. Dig Dis Sci.

